# Bihormonal fully closed-loop glucose control in adolescents with Type 1 diabetes: an outpatient, randomized, crossover trial

**DOI:** 10.48130/pedi-0026-0009

**Published:** 2026-09-02

**Authors:** Richell S. Booijink, Job S. de Haan, Loes Van Den Berg, Theodorus J.P. Jansen, Floor S. Neijens, Arianne C. Van Bon

**Affiliations:** 1 Inreda Diabetic, 7472 DD Goor, the Netherlands; 2 Department of Pediatrics, Rijnstate Hospital, 6815 AD Arnhem, the Netherlands; 3 Department of Internal Medicine, Rijnstate Hospital, 6815 AD Arnhem, the Netherlands

**Keywords:** Type 1 diabetes, Bihormonal, Pediatric, Fully closed loop

## Abstract

Effective diabetes management in adolescents with Type 1 diabetes is crucial to reduce the risk of long-term complications. However, adolescents show the poorest glycemic outcomes of all age groups. A bihormonal fully closed-loop (FCL) system, which requires no user input, may help address these challenges. In this trial, the performance and safety of a bihormonal FCL system (Inreda® AP) was evaluated in adolescents. The trial was a single-center, randomized, crossover trial in adolescents with Type 1 diabetes aged 12−18 years. The FCL system was compared with the participants' usual diabetes treatment in a home healthcare setting. The trial consisted of two study periods of 2 weeks, separated by a 2-week washout period. The primary endpoint was time in range (TIR; 3.9−10.0 mmol/L). In total, 20 individuals were enrolled and the data from 15 of them were analyzed. Glucose outcome parameters showed significant improvements during the FCL treatment period. Median TIR was 61.3% [interquartile range (IQR): 53.7%−68.2%] in the control period compared with 74.9% (IQR: 73.1−81.0) during the FCL period (*p* < 0.001). Additionally, other glucose outcomes including time above range (> 10.0 mmol/L) and median blood glucose were significantly improved in the FCL treatment period. To conclude, this trial provides preliminary evidence that treatment with the bihormonal FCL system may improve glucose outcome parameters, with no safety concerns observed in adolescents with Type 1 diabetes over the short study duration. However, future research should focus on long-term trials with larger study populations to confirm these findings and evaluate the long-term effects of bihormonal FCL treatment in adolescents.

## Introduction

Type 1 diabetes is the third most common chronic illness in adolescents (< 25 years of age)^[1]^. Good glycemic control is necessary to prevent or delay long-term diabetes related complications^[2]^. However, adolescents with Type 1 diabetes display the poorest glycemic outcomes among all age groups^[3,4]^. Guidelines from the American Diabetes Association (ADA) and the International Society for Pediatric and Adolescent Diabetes (ISPAD) recommend a target glycated hemoglobin (HbA1c) – a measure of long-term blood glucose levels – of < 7.0% (< 53 mmol/mol)^[5,6]^. In addition, ISPAD treatment goals include a time in target range (TIR; 3.9−10.0 mmol/L) of > 70%, a time below range (TBR; < 3.9 mmol/L) of <4%, and a time above range (TAR; > 10.0 mmol/L) of < 25%^[5]^. Unfortunately, only a small percentage of adolescents with Type 1 diabetes achieve these treatment goals. Poor adherence to treatment is considered to be the leading cause of treatment failure in this age group. This is influenced by factors such as education, transitioning to adult healthcare, cognitive ability, therapy complexity, and the perception of the disease^[7]^. In addition, because of pubertal hormonal changes adolescents experience increased insulin resistance^[8]^ and higher glycemic variability^[9]^. However, significant gaps in the current understanding of the pathophysiology of Type 1 diabetes in adolescents exist.

The SEARCH in Diabetes in Youth Study reported a deterioration in average HbA1c values from 8.5% (69 mmol/mol) in 2002−2007 to 9.1% (76 mmol/mol) in 2014−2019^[10]^. However, the Type 1 Diabetes Exchange Quality improvement Collaborative (T1Dx-Q1) reported a decrease in HbA1c of 0.6% in young adults (aged 16−25) in 2021−2022 compared with 2016−2017^[4]^. Similarly, the prevalence of glycemic control (mean HbA1c < 7% (< 53 mmol/mol)) increased from 7% to 19% in a comparison of the 2009−2011 with the 2021−2023 study period^[11]^. This can be partly explained by the implementation of advanced technologies for insulin delivery and blood glucose monitoring in standard diabetes care^[11].^

Automated insulin delivery (AID) systems, which combine continuous subcutaneous insulin infusion (CSII) and continuous glucose monitors (CGM) with partially automated insulin administration, have consistently increased TIR in both adults and youth without increasing the hypoglycemia risk^[12−16]^. The most broadly implemented AID systems are hybrid closed-loop (HCL) systems. These HCL systems require input from users in the form of meal announcements and activity-related adjustments, which can be challenging for children and adolescents^[17]^.

Fully closed-loop (FCL) systems are designed to eliminate the need for user input. The Inreda AP® (Inreda Diabetic, The Netherlands) is a bihormonal FCL system that provides glycemic control through the automated administration of both insulin and glucagon, guided by sensor readings^[18,19]^. In addition to lowering blood glucose levels through subcutaneous insulin infusion, this system also aids in raising blood glucose levels through subcutaneous glucagon administration in the case of (impeding) hypoglycemic events^[20]^. In adults, this system has proven its potential in various clinical trials^[18−21]^. Most recently, a 1-year trial comparing the FCL system with usual care demonstrated an increase in mean TIR of nearly 25%, as well as significantly reduced self-reported diabetes distress^[21]^.

Despite the demonstrated safety and performance of the FCL system in adults, its performance in adolescents cannot be assumed to be equivalent. Adolescence is associated with poorer glycemic control, increased glycemic variability, and varying levels of treatment adherence and self-management skills. These factors may influence the glucose dynamics and performance of the FCL system.

Therefore, in this trial, we examined the performance and safety of the FCL system in adolescents aged 12−18 during a 2-week randomized crossover trial in a home healthcare environment. The primary performance outcome was time spent within target range (3.9−10.0 mmol/L).

## Materials and methods

### Study design and participants

This trial was a single-center, randomized, unblinded, two-period, crossover trial with a 1:1 allocation ratio, designed to determine superiority of FCL treatment versus the control treatment. Randomization used computer-generated blocks. Blinding was not possible because of the nature of the intervention under investigation. There was no patient or public involvement in the design, conduct, or reporting of this trial. Study participants were recruited from the outpatient clinic of Rijnstate Hospital (Arnhem, The Netherlands). Adolescents (12−18 years) with Type 1 diabetes treated with insulin for at least 6 months were included. Exclusion criteria were impaired awareness of hypoglycemia (score ≥ 4) according to the Gold^[22]^ and/or Clarke^[23]^ questionnaire; body mass index (BMI) ≥ 35 kg/m^2^; HbA1c > 11% (97 mmol/mol); pregnancy and/or breastfeeding; use of acetaminophen; limited ability to see, hear or feel the alarm signals of the FCL system; unwillingness to act in response to the alarm signals; living alone during the night during the FCL treatment; and expected poor internet connectivity. The trial was carried out in accordance with the Declaration of Helsinki and in compliance with national regulations. It was approved by the responsible ethics committee and national competent authorities, and is registered at ClinicalTrials.gov (identifier: NCT05543850).

### Study procedures

The trial compared 2 weeks of bihormonal FCL treatment with 2 weeks of the usual diabetes treatment, which varied across multiple daily injections (MDIs) and CSII or HCL systems, with a washout period of a minimum of 2 weeks in between. A detailed description of the bihormonal FCL system and its use were published previously^[21]^. Since completion of the trial, only minor software updates have been implemented on the FCL system, which do not affect the outcomes of this study. After providing informed consent, participants were randomized to start with either of the two study periods. Because the trial was unblinded, the study personnel became aware of the assigned sequence after allocation. The 2-week FCL treatment was preceded by a training period which consisted of 2 days at the outpatient clinic to train the participants and their parents in operating the FCL system, and 4−6 days of active coaching and monitoring to optimize the FCL settings. During the FCL treatment period, the participants' glucose levels were monitored twice a day, and they were contacted after 3−6 days to discuss any problems. During the control period, participants continued their usual care, and were also contacted after 3−6 days to discuss any problems.

A blinded CGM was worn during the trial, which the participants were instructed to calibrate daily. However, calibration was not done consistently, leading to substantial deviations between the CGM measurements and the actual glucose levels, making these data inaccurate and unreliable. Therefore, it was chosen to not use the blinded CGM for calculating glucose metrics. Instead, the participants own CGM was used during the control period and the built-in CGM of the FCL system was used during the FCL treatment.

### Outcomes

The main study endpoint was the TIR (3.9−10 mmol/L; 70−180 mg/dL), calculated over both study periods. Key secondary glycemic endpoints included time in tight range (TITR; 3.9−7.8 mmol/L or 70−140 mg/dL), TBR (< 3.9 mmol/L; < 70 mg/dL), TAR (> 10.0 mmol/L; > 180 mg/dL), and the median glucose concentration. Additional glucose control metrics analyzed included TBR Level 2 (TBR2; < 3.0 mmol/L; < 54 mg/dL), TBR Level 1 (TBR1; 3.0−3.9 mmol/L; 54−70 mg/dL), TAR Level 1 (TAR1; 10.0−13.9 mmol/L; 180−250 mg/dL), TAR Level 2 (TAR2; > 13.9 mmol/L; > 250 mg/dL), glycemic variability expressed as the coefficient of variation (CV), interquartile range (IQR), and standard deviation (SD). All glucose metrics were calculated across the entire monitoring period, as well as separately for daytime (06:00−24:00) and nighttime (24:00−06:00).

Given that adolescents often, at least partially, rely on their parents for diabetes management, several questionnaires were completed by both the adolescents participating in the trial as well as their parents to evaluate person-reported outcome measures (PROMs). The questionnaires assessed were the Insulin Dosing Systems: Perceptions, Ideas, Reflections and Expectations survey (INSPIRE; range: 0−100, where lower scores indicate worse perceptions and experiences with the diabetes treatment system), the Diabetes Treatment Satisfaction Questionnaire (DTSQ, status version; range: 0−60, where higher scores reflect increased treatment satisfaction), and a shortened Technology Acceptance Scale (TAS; range: 5−25, where higher scores indicating increased technology acceptance). In addition to this, DTSQ status subscale scores were calculated as follows: Perceived diabetes control (range: 0−12, where higher scores indicating better perceived diabetes control) and perceived hypoglycemia and hyperglycemia (single questions, higher scores indicate increased perceived hypo- or hyperglycemia).

Furthermore, throughout the trial, all adverse events reported by participants were recorded and assessed systematically during each visit by the research staff. Other study parameters collected included demographic variables, weight, length, HbA1c, relevant medical history, current medication use, and insulin and glucagon use during the trial.

### Statistical analyses

Sample size was calculated using G*Power 3.1.9.4 based on a previous trial^[19]^. This resulted in a calculated sample size of 16 study participants (statistical power = 80%, significance level = 0.05). To account for potential dropout, the total sample size was increased to 20. The training period was excluded from the data analysis. Possible crossover effects were evaluated by analyzing the sums and differences of all study parameters between the two study periods. No statistically significant differences were identified in either the sum or the difference between the FCL treatment and control period, indicating the absence of carryover or period effects. Participants who completed at least 7 days of data collection in both study periods were included in the primary analysis. As a sensitivity analysis, a descriptive intention-to-treat analysis including all randomized participants was performed. However, because of substantial missing data from excluded participants, no formal statistical analysis could be performed. The distribution of the data was assessed with a histogram and Shapiro–Wilk normality test, after which a paired Student's *t*-test or Wilcoxon signed-rank test was performed to compare the data from the FCL treatment period and the control period. Normally distributed data are presented as the mean ± SD, and nonnormally distributed data are presented as the median, with the IQR in parentheses. Statistical analyses were performed according to a hierarchical testing strategy. The primary endpoint was tested using a significance level of *α* = 0.05 without multiplicity adjustment. For a set of key secondary glycemic endpoints (TITR, TBR, TAR, and median glucose concentration), a Bonferroni correction for multiple comparisons was applied, resulting in a significance threshold of *α* = 0.05/4 = 0.0125. All other glycemic and person-reported outcomes were considered to be exploratory and are reported using nominal *p*-values without formal adjustment for multiple comparisons. Data and statistical analysis was performed using Python version 3.9.17.

## Results

### Participant characteristics

Between October 2022 and January 2023, 21 participants were screened and 20 were randomized and included in the trial. One participant discontinued the FCL treatment period prematurely, and four were excluded from the analysis because of the unavailability of CGM data during the control period. The participant flow can be observed in Supplementary Fig. S1.

Data from 15 participants were included in the final analysis. Their mean age was 14.1 ± 1.5 years, the mean BMI was 20.1 ± 2.8 kg/m^2^, the mean HbA1c was 7.1 ± 1.5% (58.3 ± 7.1 mmol/mol), and 10 participants were male. Eleven participants used HCL systems for their regular diabetes care, two were using CSII, and two used MDI (Table 1).

**Table 1:** Baseline characteristics

Characteristic	Participants (*n* = 15)
Age (years)	14.1 ± 1.5
Gender	
Male	10 (66.7%)
Female	5 (33.3%)
BMI (kg/m^2^)	20.1 ± 2.8
HbA1c (%; mmol/mol)	7.1 ± 1.5; 58.3 ± 7.1
Current treatment	
HCL	11 (73.3%)
CSII	2 (13.3%)
MDI	2 (13.3%)
Current sensor	
Guardian 4	9 (60.0%)
Dexcom G6	4 (26.7%)
FreeStyle Libre 2	2 (13.4%)
BMI, body mass index; HCL, hybrid closed-loop; CSII, continuous subcutaneous insulin infusion; MDI, multiple daily injections.	

### Glucose outcomes

The glucose outcomes during the FCL treatment period and the control period are listed in Table 2. In addition, the glucose outcomes for each individual are listed in Supplementary Table S1 and the glucose outcomes per control group (FCL vs HCL, FCL vs CSII, and FCL vs MDI) are listed in Supplementary Table S2.

**Table 2:** Outcome measures of the FCL treatment period and the control period.

Outcome measure	FCL treatment period	Control period	*p-*value
Time in range (TIR; 3.9−10 mmol/L)	74.9% (73.1%−81.0%)	61.3% (53.7%−68.2%)	**< 0.001**
Daytime*	68.1% (65.1%−75.0%)	57.7% (52.3%−64.9%)	0.002
Nighttime**	95.0% (93.0%−96.2%)	74.7% (61.4%−82.5%)	< 0.001
Time in tight range (TITR; 3.9−7.8 mmol/L)	54.3% (50.7%−58.7%)	39.8% (33.4%−45.1%)	**0.001**
Daytime	44.9% (42.4%−49.8%)	36.7% (31.6%−42.2%)	0.012
Nighttime	79.0% (74.6%−81.5%)	52.8% (42.0%−56.2%)	< 0.001
Time below range (TBR; < 3.9 mmol/L)	0.8% (0.4%−1.7%)	2.1% (0.9%−3.3%)	0.022
Daytime	0.9% (0.4%−2.0%)	2.2% (1.1%−4.0%)	0.015
Nighttime	0.5% (0.1%−0.9%)	1.2% (0.0%−1.8%)	0.208
Time below range (TBR; < 3.9 mmol/L)	0.8% (0.4%−1.7%)	2.1% (0.9%−3.3%)	0.022
Daytime	0.9% (0.4%−2.0%)	2.2% (1.1%−4.0%)	0.015
Nighttime	0.5% (0.1%−0.9%)	1.2% (0.0%−1.8%)	0.303
Time below range level 2 (TBR2; < 3.0 mmol/L)	0.0% (0.0%−0.2%)	0.3% (0.1%−1.0%)	0.048
Daytime	0.1% (0.0%−0.2%)	0.3% (0.1%−0.9%)	0.048
Nighttime	0.0% (0.0%−0.0%)	0.2% (0.0%−0.4%)	0.139
Time above range (TAR; > 10.0 mmol/L)	24.5% (17.5%−25.9%)	36.2% (26.8%−45.0%)	**0.001**
Daytime	31.3% (23.3%−33.3%)	41.4% (32.6%−45.1%)	0.004
Nighttime	4.3% (2.9%−5.5%)	24.0% (13.1%−37.2%)	< 0.001
Time above range Level 1 (TAR1; 10.0−13.9 mmol/L)	16.7% (15.1%−19.7%)	22.8% (21.8%−27.2%)	0.001
Daytime	21.8% (19.7%−25.5%)	24.7% (22.8%−29.0%)	0.035
Nighttime	3.5% (2.5%−5.0%)	17.1% (11.5%−21.0%)	< 0.001
Time above range Level 2 (TAR2; > 13.9 mmol/L)	4.7% (3.1%−7.8%)	10.5% (7.0%−15.9%)	0.001
Daytime	6.1% (4.2%−10.2%)	13.1% (9.1%−17.3%)	0.001
Nighttime	0.0% (0.0%−1.1%)	2.7% (1.6%−8.6%)	0.001
Median blood glucose concentration (mmol/L)	7.4 (7.2%−7.7%)	8.5 (8.0%−9.6%)	**0.001**
Daytime	8.1 (7.6%−8.3%)	9.2 (8.3%−9.4%)	0.004
Nighttime	6.5 (6.4%−6.7%)	7.7 (7.5%−8.4%)	< 0.001
Glycemic variability			
Standard deviation (SD; mmol/L)	2.9 (2.7%−3.2%)	3.2 (3.1%−4.0%)	0.001
Coefficient of variation (CV)	35.7% (32.9%−39.0%)	38.8% (34.5%−41.6%)	0.121
Interquartile range (IQR; mmol/L)	3.7 (3.2%−3.9%)	4.8 (4.3%−5.5%)	< 0.001
Daily insulin use (units)	57.3 (52.6%−68.7%)	46.6 (37.4%−55.3%)	< 0.001
Daily glucagon use (mg)	0.55 (0.37%−0.65%)	−	
* Daytime was defined as 06:00−24:00, ** Nighttime was defined as 24:00−06:00. Data are expressed as the median (IQR). TIR was the primary endpoint, tested at a significance threshold of *α* = 0.05. TITR, TBR, TAR, and median glucose were the key secondary endpoints, evaluated using a Bonferroni-adjusted significance threshold of *α* = 0.0125. All other metrics were considered exploratory and are reported with nominal *p*-values. Statistically significant results after correction for multiple comparisons are indicated in bold. *n* = 15.			

During the FCL treatment period, the median TIR was 74.9% (73.1%−81.0%), a significant increase compared with the median TIR during the control period: 61.3% (53.7%−68.2%). In addition, TITR significantly improved in the FCL treatment period compared with the control period (54.3% [50.7−58.7] versus 39.8% [33.4−45.1]).

TBR was reduced during the FCL treatment compared with the control treatment (0.8% [0.4−1.3] versus 2.1% [0.9−3.3]), which was more pronounced during the day (0.9% [0.4−2.0] versus 2.2% [1.1−4.0]). TAR was significantly reduced (24.5% [17.5−25.9] versus 36.2% [26.8−45.0]). The proportion of time spent in the different glycemic ranges during the FCL treatment period and the control period is provided in Fig. 1.

**Figure 1:**
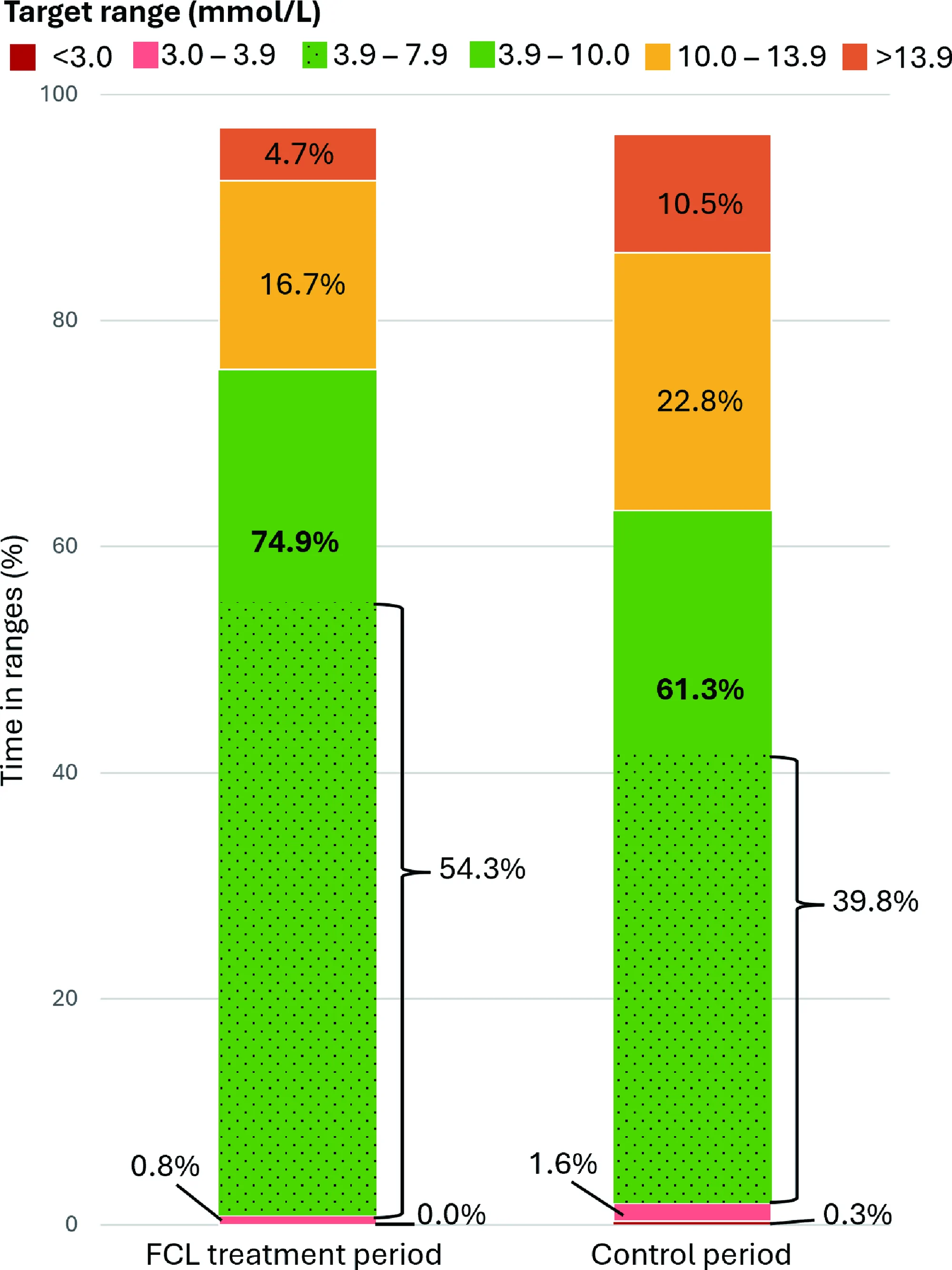
Proportion of time spent in the different glycemic ranges during the FCL treatment period and the control period. The percentages displayed are the calculated median value per category. As a result, percentages may not total to 100% as a result of skewed distributions and rounding.

The median glucose concentration was 7.4 (7.2−7.7) mmol/L during the FCL treatment period, compared with 8.5 (8.0−9.6) mmol/L during the control period. In terms of glycemic variability, SD and IQR were both reduced during the FCL treatment period, 3.2 (3.1−4.0) versus 2.9 (2.74−3.2) mmol/L and 4.8 (4.3−5.5) versus 3.7 [3.2−3.9] mmol/L, respectively. The CV, on the other hand, did not differ between the study periods.

These glucose outcome results were consistent with a descriptive intention-to-treat sensitivity analysis including all randomized participants (Supplementary Table S3).

Daily insulin use was higher during the FCL treatment period compared with the control period (46.6 [37.4−55.3] versus 57.3 [52.6−68.7] units), and daily glucagon use during the FCL treatment period was 0.55 [0.37−0.65] mg.

### Treatment expectations, trust, and satisfaction

Participants and their parents were asked to fill out several questionnaires to assess their treatment expectations, trust and satisfaction (Table 3). The DTSQ indicated that parents perceived improvements in diabetes control and a reduction in perceived hypo- and hyperglycemia in their children during the FCL treatment. In contrast, the adolescents' total DTSQ score was improved in the FCL treatment period compared with the control period, whereas improvements in perceived diabetes control or perceived reduction in hyperglycemia were only marginal. The INSPIRE questionnaire showed no differences, whereas scores on the TAS questionnaire indicated an improvement among parents during the FCL treatment period, but no corresponding change among adolescents.

**Table 3:** Person-reported outcome measures assessing treatment expectations, trust, and satisfaction.

Questionnaire	FCL treatment period	Control period	*p-*value
DTSQs: Parents	40.5 (38.0–45.0)	35.0 (28.5–39.0)	0.083
Perceived diabetes control	9.0 (6.3–10.0)	5.0 (3.5–6.0)	0.028
Perceived hypoglycemia	2.0 (1.3–2.8)	3.0 (2.0–4.0)	0.028
Perceived hyperglycemia	2.0 (1.3–4.8)	5.0 (4.0–5.0)	0.047
DTSQs: Adolescents	38.0 (35.0–44.0)	35.0 (27.0–39.0)	0.042
Perceived diabetes control	9.0 (6.0–10.0)	5.0 (4.0–8.0)	0.077
Perceived hypoglycemia	1.0 (1.0–2.0)	3.0 (2.0–3.0)	0.027
Perceived hyperglycemia	2.0 (1.0–4.0)	4.0 (3.0–5.0)	0.054
INSPIRE: Parents	81.0 (69.0–87.0)	72.0 (65.5–6.5)	0.157
INSPIRE: Adolescents	73.0 (69.0–75.0)	75.0 (66.0–82.0)	0.339
TAS: Parents	21.5 (20.0–23.0)	19.0 (17.0–21.0)	0.005
TAS: Adolescents	22.0 (19.0–23.0)	20.0 (19.0–21.0)	0.893
DTSQs, Diabetes Treatment Satisfaction Questionnaire status; INSPIRE, Insulin Delivery Systems: Perceptions, Ideas, Reflections and Expectations; TAS, Technology Acceptance Scale. Data are expressed as the median (IQR). Sample sizes: Parents (*n* = 11); adolescents (*n* = 13).			

### Adverse events

All adverse events (AEs) reported during this trial are shown in Table 4. There were no severe hypoglycemic events and no cases of diabetic ketoacidosis reported during the trial. The causes of AEs were categorized as side effects of the glucose sensors, insulin infusion set, glucagon, or FCL treatment. Most of the AEs were reported during the FCL training period preceding the FCL treatment period, and were associated with skin irritations or pruritus at the site of the sensors or infusion sets. These are common side effects of insulin pump systems^[24]^. Other AEs reported were headaches, abdominal pain, and nausea during the first week of the FCL treatment. All AEs reported were classified as mild. During the control period, no AEs were reported.

**Table 4:** Overview of all reported adverse events

Adverse events, categorized	FCL training period	FCL treatment period
Side effects of sensors		
Skin reactions, infections or damage	5	2
Side effects of insulin infusion set		
Skin reactions, infections or damage	4	−
Side effects of glucagon		
Skin reactions, infections or damage at infusion site	7	−
Nausea	4	−
Vomiting	1	−
Abdominal pain	2	−
Headache	3	1
Side effects of FCL treatment		
(Sensation of) hypoglycemia	1	−
General malaise	1	−

## Discussion

To our knowledge, this is the first study to evaluate a bihormonal FCL system in adolescents with Type 1 diabetes. Over the course of the 2-week study period, on average, the participants gained an additional 3 h per day spent in normal glycemic range during the FCL treatment. This improvement is largely attributed to a decrease in TAR. Moreover, significant improvements in TBR and all other glucose outcomes were also observed. Furthermore, during the bihormonal FCL treatment, the therapy goals outlined in the ISPAD clinical guidelines were met on average, and all participants achieved a TIR > 70%.

Given the well-known problems that adolescents experience with treatment adherence^[7]^, this group in particular may benefit from treatment with an FCL system. Consistent with this, one study reported that more automated diabetes management was associated with more consistent self-management behaviors in adolescents, and resulted in fewer self-regulation failures and lower mean daily blood glucose^[25]^. Despite this, there are only limited other published studies examining the use of FCL systems in adolescents. A previous study from 2008 compared an FCL system with an HCL system using a pre-meal priming bolus for 34 h. Higher mean glucose levels were observed during treatment with the FCL system, suggesting that single-hormone FCL systems were not yet sufficient for optimal glucose control^[26]^. However, meal announcement technologies have improved significantly in recent years. For example, a study of adolescents and young adults found that glucose levels remained similar when comparing simplified meal announcements with carbohydrate counting^[27]^. Moreover, a recent study investigated FCL therapy (CamAPS HX) with faster insulin aspart in adolescents using insulin pump therapy with above-target HbA1c of 8.9% (74 mmol/mol), which was considerably higher than the 7.1% (58 mmol/mol) seen in our cohort. Over an 8-week period, FCL treatment resulted in a 12.9 percentage point increase in TIR compared with insulin pump treatment with CGM (45.4% ± 7.2 versus 32.3% ± 12.8)^[28]^. However, the 45% TIR reported in that study remained well below the guideline-recommended target of > 70%, and was lower than the TIR observed in both the control and FCL treatment period in our research. This suggests that our study population was better regulated at baseline, limiting direct comparisons between studies.

Questionnaires were completed by both the adolescents and their parents, as parents are often extensively involved in the diabetes management of their child. PROMs demonstrated more heterogeneous results, with the benefits of the FCL system not observed across all assessed domains. For adolescents, total DTSQ score was improved and the perceived hypoglycemia score lowered, indicating that the participants experienced improvements in diabetes management during the FCL treatment. However, no clear improvement was observed in the TAS score (indicative of the level of trust in the technology used) or in the INSPIRE score, which was designed to evaluate perceptions and experiences with the insulin delivery system. These findings were consistent with unstructured feedback provided by participants to the investigators after the trial. They mentioned benefits of the FCL system that included feeling more energized, an increased ability to concentrate, and an increased sense of freedom, which are indicative of improved diabetes control. On the other hand, the quantity of alarms, daily replacement of glucagon, and the large size of the FCL system were considered disadvantages. Similar benefits and disadvantages have recently been noted in adult users of the same device. Moreover, users' feedback and safety experiences have allowed some alarm modifications in the most recent software version of the device^[29]^. Parents' scores showed slightly different results. Total DTSQ score was not improved, although parents perceived less hypoglycemia and hyperglycemia in the glucose regulation of their children, as well as improved diabetes control. Interestingly, the parents' TAS score was improved in the FCL treatment period. This suggests that parents have more trust in the FCL system compared with their child's regular diabetes treatment. This is supported by feedback after the trial, where parents reported a reduced urge to check on their child's diabetes management when they used the FCL system. However, it should be noted that the study was unblinded, which may have influenced the outcomes of the questionnaires and interviews of both the adolescents and their parents.

The incorporation of glucagon into the FCL system may contribute to tighter glucose regulation. Subcutaneous administration of glucagon facilitates an increase in blood glucose concentrations in response to impeding hypoglycemia, thereby enhancing glycemic stability^[30]^. In the current trial, median daily glucagon use was 0.55 mg, which was comparable with that of adults using the same device in a prospective trial for 1 year^[21]^. Median insulin use increased substantially in the FCL treatment period compared with the control period. This was expected, as the bihormonal aspect of the FCL system allows for higher insulin doses to be administered safely because insulin-induced hypoglycemia can be counteracted by glucagon. Moreover, the FCL system provides fully automated insulin and glucagon dosing based on the most recently measured CGM values, without any user input or preset basal profiles, acting upon the current situation. Thus, insulin administration relies on a different mechanism compared with the control treatments, which are – at least partially – user-initiated. The increased insulin dose during the FCL period contributed to improved glycemic control, as indicated by a higher TIR and lower TAR. Median insulin dose during the FCL treatment period was higher in adolescents compared with a previous trial with adults^[21]^, which may be attributed to the increased insulin requirements observed during adolescence compared with adulthood^[31]^.

Most AEs were reported during the FCL training period, with few AEs during the FCL treatment period and none during the control period. This pattern likely reflects underreporting during the main study period, as AE collection was carried out during the study visits that followed after each study period and therefore relied on the recollection of AEs experienced by the participants. During the training period, AEs were actively monitored. Moreover, there may have been some bias in AE reporting due to the unblinded nature of the trial. All events were classified as mild, and most are well-known AEs that also frequently occur in other pump systems, including skin reactions from the glucose sensors and infusion sets^[24]^. However, the FCL uses two sensors and two infusion sets, one for insulin and one for glucagon, which increases the risk of skin irritations. This should be considered for children and adolescents in particular, as skin problems can be a reason for discontinuation of the treatment^[24]^. Other AEs reported were related to glucagon use, including headaches, nausea and abdominal pain, which are known side effects of glucagon. Similar events were reported in a 1-year trial in adults using the same bihormonal FCL device, where these glucagon-related AEs substantially decreased over time, suggesting habituation to glucagon-related effects may apply if the FCL system is used for a longer period^[21]^.

Several limitations related to the study design should be acknowledged. One limitation is that for glycemic data collection during the control period, the patients' own CGM systems were used, whereas during the FCL period, the built-in CGM of the FCL system was used. This potential variation in CGM performance across devices may have introduced bias in the comparison of glycemic outcomes, including TIR. As a result, the precision of the comparison between study periods might have been impacted. However, considering the large differences in glycemic outcomes between the two study periods, we assume that this bias did not significantly impact the main conclusions of this study. A second limitation is the heterogeneity of the control group, which consisted of participants using MDI, CSII, and HCL as their regular diabetes treatment. This may have reduced the precision of estimating the effect of the FCL treatment. Another limitation is that this trial had a relatively short observation period of 2 weeks per treatment. This is insufficient to assess the long-term effects of the FCL system and the stability of the glucose outcomes. This is particularly relevant in adolescents, where treatment adherence is known to be challenging. Longer-term future studies are therefore needed to assess these findings over extended follow-up periods, making a comparison between current commercially available treatment options and bihormonal FCL treatment.

## Conclusions

This trial provides preliminary evidence that treatment with the bihormonal FCL system may improve glucose outcomes, with no safety concerns observed in adolescents with Type 1 diabetes over the short study duration. Future research should focus on long-term trials with larger study populations to confirm these findings and evaluate the long-term effects of bihormonal FCL treatment in adolescents.

## SUPPLEMENTARY DATA

Supplementary data to this article can be found online.

## Data Availability

The data that support the findings of this trial are available from Inreda Diabetic B.V., but restrictions apply to the availability of these data. Therefore, these data are not publicly available. Data are, however, available from the authors upon reasonable request and with the permission of Inreda Diabetic B.V.
