# Supplementary material for: Bihormonal fully closed-loop glucose control in adolescents with Type 1 diabetes: an outpatient, randomized, crossover trial
Source: Pediatr Diabetes. 2026 Sep 2;2026:e008. doi: 10.48130/pedi-0026-0009 (PMC13599424; doi:10.48130/pedi-0026-0009)
Supplement: Supplementary file 1 [file pedi-2026-2026-1-0009-S1.zip › 10.48130_pedi-0026-0009-Suppl-TableS1.docx]

**Supplementary Table 1. Glucose outcomes of the FCL treatment period and control period per participant**

|  | **Treatment** | **TBR2** | **TBR1** | **TBR** | **TIR** | **TITR** | **TAR** | **TAR1** | **TAR2** | **TBR2 day** | **TBR1 day** | **TBR day** | **TIR day** | **TITR day** | **TAR day** | **TAR1 day** | **TAR2 day** | **TBR2 night** | **TBR1 night** | **TBR night** | **TIR night** | **TITR night** | **TAR night** | **TAR1 night** | **TAR2 night** | **Median Blood Glucose** | **SD** | **CV** | **IQR** | **Median Blood**  **Glucose night** | **Median Blood**  **Glucose day** |
| --- | --- | --- | --- | --- | --- | --- | --- | --- | --- | --- | --- | --- | --- | --- | --- | --- | --- | --- | --- | --- | --- | --- | --- | --- | --- | --- | --- | --- | --- | --- | --- |
| 1 | FCL treatment | 0.4 | 1.7 | 2.1 | 82.1 | 61.7 | 15.8 | 12.8 | 3.0 | 0.2 | 1.6 | 1.8 | 79.1 | 56.9 | 19.1 | 15.2 | 3.9 | 1.1 | 2.2 | 3.3 | 91.6 | 77.4 | 5.1 | 5.1 | 0.0 | 7.0 | 2.6 | 34.8 | 2.9 | 6.4 | 7.3 |
|  | Control | 0.5 | 1.1 | 1.6 | 58.5 | 39.6 | 39.9 | 22.1 | 17.8 | 0.6 | 1.5 | 2.1 | 53.2 | 32.7 | 44.7 | 24.4 | 20.3 | 0.0 | 0.0 | 0.0 | 76.0 | 62.6 | 24.0 | 14.6 | 9.3 | 8.7 | 4.1 | 41.4 | 5.6 | 7.2 | 9.4 |
| 2 | FCL treatment | 0.0 | 0.7 | 0.7 | 73.5 | 55.6 | 25.8 | 20.2 | 5.7 | 0.0 | 0.7 | 0.7 | 66.2 | 45.7 | 33.1 | 25.9 | 7.1 | 0.0 | 0.4 | 0.4 | 95.6 | 85.5 | 4.0 | 2.7 | 1.3 | 7.4 | 3.0 | 35.6 | 4.1 | 6.4 | 8.1 |
|  | Control | 1.1 | 2.7 | 3.8 | 50.0 | 23.4 | 46.2 | 35.7 | 10.5 | 1.4 | 3.6 | 5.1 | 51.7 | 27.8 | 43.2 | 30.5 | 12.8 | 0.0 | 0.0 | 0.0 | 44.8 | 10.3 | 55.2 | 51.4 | 3.8 | 9.8 | 3.4 | 35.2 | 4.2 | 10.3 | 9.4 |
| 3 | FCL treatment | 0.0 | 0.3 | 0.3 | 78.1 | 55.0 | 21.6 | 16.7 | 4.9 | 0.0 | 0.4 | 0.4 | 73.5 | 47.1 | 26.2 | 19.8 | 6.4 | 0.0 | 0.1 | 0.1 | 91.4 | 77.9 | 8.5 | 7.6 | 0.9 | 7.4 | 2.9 | 35.9 | 3.4 | 6.5 | 8.0 |
|  | Control | 0.2 | 0.7 | 0.9 | 53.0 | 24.6 | 46.1 | 28.2 | 17.9 | 0.2 | 0.7 | 0.9 | 40.0 | 16.6 | 59.1 | 35.4 | 23.7 | 0.0 | 1.0 | 1.0 | 88.5 | 46.4 | 10.5 | 8.5 | 2.0 | 9.7 | 3.7 | 35.2 | 5.0 | 8.0 | 11.0 |
| 4 | FCL treatment | 0.0 | 0.8 | 0.8 | 86.6 | 59.7 | 12.6 | 11.5 | 1.0 | 0.0 | 0.8 | 0.8 | 83.7 | 55.2 | 15.5 | 14.1 | 1.4 | 0.0 | 0.9 | 0.9 | 95.4 | 73.4 | 3.7 | 3.7 | 0.0 | 7.3 | 2.2 | 28.5 | 2.8 | 6.7 | 7.5 |
|  | Control | 0.2 | 0.8 | 0.9 | 72.7 | 45.8 | 26.3 | 22.4 | 3.9 | 0.2 | 1.0 | 1.3 | 68.8 | 42.2 | 30.0 | 25.1 | 4.8 | 0.0 | 0.0 | 0.0 | 84.2 | 56.3 | 15.8 | 14.5 | 1.3 | 8.2 | 2.8 | 32.4 | 3.7 | 7.5 | 8.4 |
| 5 | FCL treatment | 0.0 | 0.5 | 0.5 | 71.4 | 43.7 | 28.2 | 19.6 | 8.5 | 0.0 | 0.4 | 0.4 | 63.7 | 37.9 | 35.9 | 25.1 | 10.8 | 0.0 | 0.7 | 0.7 | 94.0 | 60.9 | 5.3 | 3.5 | 1.8 | 8.1 | 3.2 | 36.0 | 3.8 | 7.4 | 8.7 |
|  | Control | 1.1 | 8.1 | 9.2 | 43.3 | 21.0 | 47.6 | 36.0 | 11.6 | 1.1 | 10.3 | 11.4 | 39.8 | 23.1 | 48.8 | 34.4 | 14.4 | 1.1 | 1.1 | 2.1 | 54.2 | 14.4 | 43.7 | 41.0 | 2.7 | 9.7 | 3.9 | 40.0 | 5.1 | 9.6 | 9.8 |
| 6 | FCL treatment | 0.1 | 0.2 | 0.3 | 72.5 | 53.9 | 27.2 | 19.3 | 7.9 | 0.2 | 0.2 | 0.4 | 64.6 | 43.5 | 35.0 | 24.9 | 10.1 | 0.0 | 0.0 | 0.0 | 94.8 | 83.4 | 5.2 | 3.6 | 1.6 | 7.5 | 3.4 | 39.4 | 4.2 | 6.4 | 8.3 |
|  | Control | 0.2 | 1.4 | 1.6 | 54.5 | 36.0 | 43.9 | 22.7 | 21.2 | 0.1 | 1.5 | 1.6 | 52.9 | 32.8 | 45.5 | 24.7 | 20.9 | 0.4 | 1.3 | 1.7 | 59.1 | 45.4 | 39.2 | 17.1 | 22.1 | 9.1 | 4.5 | 43.6 | 6.5 | 8.1 | 9.4 |
| 7 | FCL treatment | 0.2 | 1.1 | 1.3 | 73.3 | 47.4 | 25.4 | 22.0 | 3.4 | 0.3 | 1.4 | 1.7 | 68.1 | 43.0 | 30.2 | 26.0 | 4.2 | 0.0 | 0.1 | 0.1 | 88.2 | 60.1 | 11.7 | 10.7 | 0.9 | 7.9 | 2.7 | 32.9 | 4.0 | 7.2 | 8.3 |
|  | Control | 0.0 | 0.3 | 0.3 | 56.6 | 31.5 | 43.1 | 31.1 | 11.9 | 0.0 | 0.4 | 0.4 | 57.7 | 36.6 | 41.9 | 28.6 | 13.3 | 0.0 | 0.0 | 0.0 | 53.5 | 16.5 | 46.5 | 38.6 | 7.9 | 9.4 | 3.3 | 34.3 | 4.8 | 9.8 | 9.2 |
| 8 | FCL treatment | 0.0 | 0.3 | 0.3 | 73.9 | 51.7 | 25.9 | 15.5 | 10.3 | 0.0 | 0.0 | 0.0 | 65.9 | 41.7 | 34.1 | 20.2 | 13.9 | 0.0 | 1.1 | 1.1 | 97.0 | 80.6 | 1.9 | 1.9 | 0.0 | 7.7 | 3.5 | 40.1 | 3.8 | 6.4 | 8.5 |
|  | Control | 0.0 | 0.0 | 0.0 | 50.4 | 35.2 | 49.6 | 26.1 | 23.5 | 0.0 | 0.0 | 0.0 | 42.7 | 30.5 | 57.3 | 29.3 | 27.9 | 0.0 | 0.0 | 0.0 | 72.9 | 49.0 | 27.1 | 16.6 | 10.5 | 10.0 | 4.2 | 39.1 | 6.7 | 8.0 | 10.9 |
| 9 | FCL treatment | 0.0 | 0.5 | 0.5 | 82.0 | 58.6 | 17.5 | 16.4 | 1.0 | 0.0 | 0.7 | 0.7 | 75.9 | 50.2 | 23.3 | 21.9 | 1.4 | 0.0 | 0.0 | 0.0 | 99.0 | 82.3 | 1.0 | 1.0 | 0.0 | 7.3 | 2.3 | 29.4 | 3.1 | 6.5 | 7.8 |
|  | Control | 0.4 | 1.6 | 2.0 | 88.6 | 68.8 | 9.4 | 9.0 | 0.4 | 0.5 | 1.8 | 2.2 | 86.9 | 66.6 | 10.9 | 10.4 | 0.5 | 0.3 | 1.2 | 1.5 | 93.9 | 76.0 | 4.6 | 4.6 | 0.0 | 6.9 | 2.1 | 29.2 | 2.3 | 6.8 | 6.9 |
| 10 | FCL treatment | 0.1 | 0.9 | 0.9 | 75.2 | 54.1 | 23.9 | 16.3 | 7.6 | 0.1 | 1.0 | 1.1 | 66.8 | 45.6 | 32.1 | 21.9 | 10.3 | 0.0 | 0.6 | 0.6 | 99.4 | 78.7 | 0.0 | 0.0 | 0.0 | 7.5 | 3.3 | 39.2 | 3.7 | 6.6 | 8.1 |
|  | Control | 0.3 | 1.9 | 2.2 | 74.5 | 49.3 | 23.4 | 19.3 | 4.1 | 0.4 | 2.5 | 2.9 | 68.3 | 45.6 | 28.9 | 23.3 | 5.5 | 0.0 | 0.1 | 0.1 | 92.6 | 59.8 | 7.3 | 7.3 | 0.0 | 7.7 | 2.9 | 34.8 | 3.9 | 7.5 | 8.0 |
| 11 | FCL treatment | 0.0 | 0.2 | 0.2 | 75.0 | 50.8 | 24.8 | 20.9 | 3.8 | 0.0 | 0.3 | 0.3 | 68.5 | 42.2 | 31.2 | 26.4 | 4.8 | 0.0 | 0.0 | 0.0 | 92.8 | 74.6 | 7.2 | 5.9 | 1.3 | 7.8 | 2.8 | 33.0 | 3.5 | 7.0 | 8.3 |
|  | Control | 0.1 | 0.3 | 0.4 | 63.5 | 39.8 | 36.2 | 25.8 | 10.3 | 0.0 | 0.0 | 0.0 | 58.6 | 35.5 | 41.4 | 28.4 | 12.9 | 0.2 | 1.1 | 1.3 | 78.5 | 53.1 | 20.2 | 17.8 | 2.4 | 8.5 | 3.1 | 33.3 | 4.4 | 7.7 | 9.2 |
| 12 | FCL treatment | 1.5 | 2.7 | 4.2 | 77.3 | 58.5 | 18.6 | 14.6 | 3.9 | 2.0 | 3.6 | 5.6 | 72.1 | 50.7 | 22.3 | 17.2 | 5.1 | 0.0 | 0.0 | 0.0 | 92.0 | 80.9 | 8.0 | 7.3 | 0.7 | 7.0 | 2.9 | 37.6 | 3.4 | 6.5 | 7.2 |
|  | Control | 0.1 | 2.3 | 2.3 | 62.1 | 41.3 | 35.6 | 21.6 | 14.0 | 0.0 | 2.7 | 2.7 | 61.6 | 42.2 | 35.7 | 22.2 | 13.6 | 0.2 | 1.0 | 1.2 | 63.6 | 38.6 | 35.2 | 19.7 | 15.5 | 8.4 | 4.1 | 43.4 | 5.5 | 8.6 | 8.3 |
| 13 | FCL treatment | 0.6 | 1.6 | 2.2 | 79.4 | 58.4 | 18.4 | 15.3 | 3.2 | 0.7 | 2.0 | 2.7 | 73.2 | 49.2 | 24.1 | 19.9 | 4.3 | 0.2 | 0.6 | 0.8 | 97.6 | 85.4 | 1.6 | 1.6 | 0.0 | 7.7 | 2.7 | 34.6 | 3.4 | 6.4 | 8.2 |
|  | Control | 1.3 | 4.0 | 5.3 | 61.3 | 42.2 | 33.4 | 23.4 | 10.1 | 1.5 | 5.0 | 6.5 | 56.7 | 38.6 | 36.9 | 24.2 | 12.6 | 0.9 | 1.0 | 1.9 | 74.7 | 52.8 | 23.5 | 20.9 | 2.6 | 8.9 | 3.9 | 43.7 | 5.4 | 8.1 | 9.1 |
| 14 | FCL treatment | 0.1 | 0.6 | 0.7 | 86.4 | 61.0 | 12.9 | 11.1 | 1.8 | 0.1 | 0.8 | 0.9 | 83.5 | 56.8 | 15.6 | 13.2 | 2.4 | 0.0 | 0.0 | 0.0 | 95.0 | 73.5 | 5.0 | 5.0 | 0.0 | 7.7 | 2.2 | 28.9 | 2.7 | 7.0 | 7.9 |
|  | Control | 0.9 | 1.9 | 2.8 | 70.8 | 54.4 | 26.4 | 22.0 | 4.4 | 1.1 | 1.9 | 3.0 | 70.9 | 54.7 | 26.1 | 22.2 | 3.9 | 0.2 | 1.9 | 2.1 | 70.6 | 53.6 | 27.2 | 21.2 | 6.1 | 8.1 | 3.1 | 38.8 | 4.5 | 8.5 | 8.0 |
| 15 | FCL treatment | 0.0 | 1.9 | 2.0 | 73.0 | 49.5 | 25.1 | 20.3 | 4.8 | 0.0 | 2.3 | 2.4 | 64.5 | 39.7 | 33.1 | 26.5 | 6.6 | 0.0 | 0.9 | 0.9 | 95.4 | 75.5 | 3.7 | 3.7 | 0.0 | 8.4 | 3.2 | 38.5 | 3.9 | 6.7 | 9.0 |
|  | Control | 1.3 | 5.9 | 7.3 | 65.5 | 44.4 | 27.2 | 17.5 | 9.6 | 0.3 | 4.7 | 5.1 | 59.8 | 40.0 | 35.1 | 22.0 | 13.1 | 4.0 | 9.2 | 13.2 | 80.8 | 56.1 | 6.1 | 5.7 | 0.4 | 8.5 | 3.5 | 41.7 | 4.3 | 6.8 | 9.1 |

Abbreviations: FCL, fully closed-loop; TBR2, time below range level 2; TBR1, time below range level 1; TIR, time in range; TITR, time in tight range; TAR1, time above range level 1; TAR2, time above range level 2; SD, standard deviation; CV, coefficient of variation; IQR, interquartile range;
Day: 06:00 – 24:00
Night: 24:00 – 06:00
Glycemic ranges and CV are presented as percentage.
Blood glucose, SD and IQR are expressed as mmol/L.
