# Supplementary material for: Bihormonal fully closed-loop glucose control in adolescents with Type 1 diabetes: an outpatient, randomized, crossover trial
Source: Pediatr Diabetes. 2026 Sep 2;2026:e008. doi: 10.48130/pedi-0026-0009 (PMC13599424; doi:10.48130/pedi-0026-0009)
Supplement: Supplementary file 1 [file pedi-2026-2026-1-0009-S1.zip › 10.48130_pedi-0026-0009-Suppl-TableS2.docx]

**Supplementary Table 2. Glucose outcomes per control group**

|  | **FCL vs HCL (n=11)** | | **FCL vs CSII (n=2)** | | **FCL vs MDI (n=2)** | |
| --- | --- | --- | --- | --- | --- | --- |
|  | FCL | HCL | FCL | CSII | FCL | MDI |
| **Time in range (TIR; 3.9 – 10 mmol/L)**  Daytime  Nighttime | 78.1 (75.1 – 82.0)  73.2 (67.7 – 77.5)  95.4 (92.4 – 97.3) | 63.5 (59.9 – 71.8)  59.8 (54.9 – 68.5)  78.5 (73.8 – 86.4) | 72.9 (72.7 – 73.1)  66.4 (65.5 – 67.2)  91.5 (89.9 – 93.2) | 55.5 (55.0 – 56.1)  55.3 – 54.1 – 56.3)  56.3 (54.9 – 57.7) | 72.4 (71.9 – 73.0)  65.0 (64.3 – 65.6)  94.8 (94.4 – 95.2) | 46.6 (45.0 – 48.3)  45.8 (42.8 – 48.8)  49.5 (47.1 – 51.8) |
| **Time in tight range (TITR; 3.9 – 7.8 mmol/L)**  Daytime  Nighttime | 58.4 (52.9 – 59.2)  49.2 (43.9 – 53.0)  77.9 (75.1 – 80.7) | 42.2 (39.7 – 47.5)  40.0 (34.1 – 43.9)  53.6 (50.9 – 58.0) | 50.6 (49.0 – 52.3)  43.2 (43.1 – 43.3)  71.7 (65.9 – 77.6) | 33.8 (32.7 – 34.9)  34.7 (33.8 – 35.7)  30.9 (23.7 – 38.1) | 49.7 (46.7 – 52.7)  41.8 (39.9 – 43.8)  73.2 (67.0 – 79.4) | 22.2 (21.6 – 22.8)  25.4 (24.3 – 26.6)  12.4 (11.3 – 13.4) |
| **Time below range (TBR; <3.9 mmol/L)**  Daytime  Nighttime | 0.8 (0.4 – 2.0)  0.9 (0.6 – 2.1)  0.6 (0.0 – 0.9) | 2.0 (0.9 – 2.6)  2.2 (1.1 – 2.9)  1.2 (0.0 – 1.7) | 0.8 (0.5 – 1.0)  1.1 (0.7 – 1.4)  0.1 (0.0 – 0.1) | 1.0 (0.7 – 1.3)  1.0 (0.7 – 1.3)  0.9 (0.4 – 1.3) | 0.6 (0.5 – 0.6)  0.6 (0.5 – 0.7)  0.5 (0.5 – 0.6) | 6.5 (5.1 – 7.8)  8.2 (6.6 – 9.8)  1.1 (0.5 – 1.6) |
| **Time below range level 1 (TBR1; 3.0 – 3.9 mmol/L)**  Daytime  Nighttime | 0.8 (0.4 – 1.7)  0.8 (0.6 – 1.8)  0.6 (0.0 – 0.9) | 1.6 (0.8 – 2.1)  1.8 (0.8 – 2.6)  1.0 (0.0 – 1.1) | 0.6 (0.4 – 0.8)  0.8 (0.5 – 1.1)  0.1 (0.0 – 0.1) | 0.9 (0.6 – 1.1)  0.9 (0.7 – 1.2)  0.7 (0.3 – 1.0) | 0.6 (0.5 – 0.6)  0.6 (0.5 – 0.7)  0.5 (0.5 – 0.6) | 5.4 (4.1 – 6.7)  7.0 (5.3 – 8.6)  0.5 (0.3 - 0.8) |
| **Time below range level 2 (TBR2; <3.0 mmol/L)**  Daytime  Nighttime | 0.0 (0.0 – 0.2)  0.0 (0.0 – 0.1)  0.0 (0.0 – 0.0) | 0.3 (0.1 – 0.7)  0.3 (0.1 – 0.6)  0.2 (0.0 – 0.3) | 0.2 (0.2 – 0.2)  0.2 (0.2 – 0.3)  0.0 (0.0 – 0.0) | 0.1 (0.1 – 0.2)  0.1 (0.0 – 0.1)  0.2 (0.1 – 0.3) | 0.0 (0.0 – 0.0)  0.0 (0.0 – 0.0)  0.0 (0.0 – 0.0) | 1.1 (1.1 – 1.1)  1.3 (1.2 – 1.4)  0.5 (0.3 – 0.8) |
| **Time above range (TAR; >10.0 mmol/L)**  Daytime  Nighttime | 18.6 (16.6 – 24.3)  24.1 (20.7 – 31.7)  3.7 (1.8 – 6.1) | 33.4 (26.4 – 38.0)  35.7 (29.4 – 43.0)  20.2 (8.9 – 25.5) | 26.3 (25.9 – 26.8)  32.6 (31.4 – 33.8)  8.4 (6.8 – 10.0) | 43.5 (43.3 – 43.7)  43.7 (42.8 – 44.6)  42.9 (41.0 – 44.7) | 27.0 (26.4 – 27.6)  34.5 (33.8 – 35.2)  4.6 (4.3 – 5.0) | 46.9 (46.5 – 47.2)  46.0 (44.6 – 47.4)  49.4 (46.6 – 52.3) |
| **Time above range level 1 (TAR1; 10.0 – 13.9 mmol/L)**  Daytime  Nighttime | 15.5 (13.7 – 16.6)  19.9 (16.2 – 21.9)  3.7 (1.8 – 5.5) | 22.1 (20.4 – 24.6)  24.2 (22.2 – 26.8)  14.6 (7.9 – 18.8) | 20.7 (20.0 – 21.4)  25.4 (25.1 – 25.7)  7.2 (5.4 – 9.0) | 26.9 (24.8 – 29.0)  26.6 (25.7 – 27.6)  27.8 (22.5 – 33.2) | 19.9 (19.8 – 20.0)  25.5 (25.3 – 25.7)  3.1 (2.9 – 3.3) | 35.8 (35.7 – 35.9)  32.4 (31.4 – 33.4)  46.2 (43.6 – 48.8) |
| **Time above range level 2 (TAR2; >13.9 mmol/L)**  Daytime  Nighttime | 3.8 (2.4 – 4.9)  4.8 (3.2 – 6.5)  0.0 (0.0 – 0.4) | 10.1 (4.3 – 15.9)  12.9 (5.2 – 16.9)  2.4 (0.8 – 7.7) | 5.6 (4.5 – 6.7)  7.2 (5.7 – 8.6)  1.2 (1.1 – 1.4) | 16.6 (14.2 – 18.9)  17.1 (15.2 – 19.0)  15.0 (11.5 – 18.6) | 7.1 (6.4 – 7.8)  9.0 (8.0 – 9.9)  1.5 (1.4 – 1.7) | 11.0 (10.8 – 11.3)  13.6 (13.2 – 14.0)  3.3 (3.0 – 3.5) |
| **Median blood glucose concentration (mmol/L)**  Daytime  Nighttime | 7.3 (7.2 – 7.6)  7.8 (7.5 – 8.2)  6.5 (6.5 – 6.7) | 8.2 (7.8 – 8.6)  8.4 (8.2 – 9.3)  7.5 (7.3 – 7.9) | 7.7 (7.6 - 7.8)  8.3 (8.3 – 8.3)  6.8 (6.6 – 7.0) | 9.3 (9.2 – 9.3)  9.3 (9.3 – 9.4)  9.0 (8.5 – 9.4) | 7.8 (7.6 – 7.9)  8.4 (8.3 – 8.6)  6.9 (6.7 – 7.2) | 9.8 (9.7 – 9.8)  9.6 (9.5 – 9.7)  10.0 (9.8 – 10.1) |
| **Glycemic variability**  Standard deviation (SD; mmol/L)  Coefficient of variation (CV)  Interquartile range (IQR; mmol/L) | 2.8 (2.5 – 3.1)  34.8 (31.2 – 38.1)  3.4 (3.0 – 3.6) | 3.5 (3.0 – 4.0)  38.8 (34.0 – 41.6)  4.5 (4.1 – 5.5) | 3.0 (2.9 – 3.2)  36.1 (34.5 – 37.7)  4.1 (4.0 – 4.2) | 3.9 (3.6 – 4.2)  38.9 (36.6 – 41.2)  5.7 (5.2 – 6.10) | 3.1 (3.0 – 3.1)  35.8 (35.7 – 35.9)  3.9 (3.8 – 4.0) | 3.7 (3.6 – 3.8)  37.6 (36.4 – 38.8)  4.7 (4.4 – 4.9) |

Abbreviations: FCL, fully closed loop; HCL, hybrid closed loop; CSII, continuous subcutaneous insulin infusion; MDI, multiple daily injections.
Day: 06:00 – 24:00
Night: 24:00 – 06:00
Data are presented as median (IQR).
Glycemic ranges and CV are presented as percentage.
Blood glucose, SD and IQR are expressed as mmol/L.
