# Supplementary material for: Bihormonal fully closed-loop glucose control in adolescents with Type 1 diabetes: an outpatient, randomized, crossover trial
Source: Pediatr Diabetes. 2026 Sep 2;2026:e008. doi: 10.48130/pedi-0026-0009 (PMC13599424; doi:10.48130/pedi-0026-0009)
Supplement: Supplementary file 1 [file pedi-2026-2026-1-0009-S1.zip › 10.48130_pedi-0026-0009-Suppl-TableS3.docx]

**Supplementary Table 3. Descriptive intention-to-treat analysis of glucose outcomes in all randomized participants.**

|  | **FCL treatment period (n=20)** | **Control period (n=17)*** |
| --- | --- | --- |
| **Time in range (TIR; 3.9 – 10 mmol/L)**  Daytime  Nighttime | 75.1 (73.2 - 82.2)  69.0 (65.2 – 77.0)  94.9 (92.6 – 96.0) | 59.9 (52.9 - 66.9)  57.2 (49.5 - 63.2)  75.3 (62.5 - 85.3) |
| **Time in tight range (TITR; 3.9 – 7.8 mmol/L)**  Daytime  Nighttime | 54.7 (50.1 - 58.7)  45.2 (41.5 - 50.6)  77.4 (72.0 - 81.2) | 39.7 (29.8 - 44.8)  36.1 (29.8 - 42.2)  50.9 (36.5 - 56.2) |
| **Time below range (TBR; <3.9 mmol/L)**  Daytime  Nighttime | 0.9 (0.4 - 1.9)  1.0 (0.4 - 1.9)  0.6 (0.0 - 0.9) | 1.8 (0.8 - 3.0)  2.2 (0.8 - 3.5)  1.1 (0.0 - 1.8) |
| **Time below range level 1 (TBR1; 3.0 – 3.9 mmol/L)**  Daytime  Nighttime | 0.9 (0.4 - 1.4)  0.9 (0.4 - 1.5)  0.6 (0.0 - 0.9) | 1.5 (0.6 - 2.4)  1.6 (0.6 - 2.9)  1.0 (0.0 - 1.1) |
| **Time below range level 2 (TBR2; <3.0 mmol/L)**  Daytime  Nighttime | 0.1 (0.0 - 0.2)  0.1 (0.0 - 0.3)  0.0 (0.0 - 0.0) | 0.3 (0.1 - 0.9)  0.3 (0.0 - 0.8)  0.1 (0.0 - 0.3) |
| **Time above range (TAR; >10.0 mmol/L)**  Daytime  Nighttime | 24.1 (16.6 - 26.0)  30.3 (21.9 – 33.0)  4.9 (3.2 - 6.0) | 38.0 (27.0 - 46.1)  41.6 (33.8 - 46.4)  23.7 (9.7 - 36.2) |
| **Time above range level 1 (TAR1; 10.0 – 13.9 mmol/L)**  Daytime  Nighttime | 16.3 (13.9 - 19.7)  21.6 (18.1 - 25.3)  4.0 (2.5 - 5.1) | 22.6 (21.0 - 26.6)  24.9 (23.1 - 28.8)  16.8 (8.2 - 20.9) |
| **Time above range level 2 (TAR2; >13.9 mmol/L)**  Daytime  Nighttime | 4.2 (2.5 - 7.7)  5.3 (3.1 - 10.2)  0.0 (0.0 - 1.2) | 11.0 (8.3 - 17.8)  13.2 (10.9 - 20.4)  2.6 (1.1 - 8.3) |
| **Median blood glucose concentration (mmol/L)**  Daytime  Nighttime | 7.4 (7.2 - 7.7)  8.1 (7.7 - 8.3)  6.5 (6.4 – 7.0) | 8.6 (8.0 - 9.6)  9.2 (8.3 - 9.5)  7.8 (7.5 - 8.2) |
| **Glycemic variability**  Standard deviation (SD; mmol/L)  Coefficient of variation (CV)  Interquartile range (IQR; mmol/L) | 2.8 (2.5 - 3.2)  35.3 (32.6 – 38.9)  3.5 (3.0 - 3.9) | 3.6 (3.1 - 4.0)  0.4 (0.3 - 0.4)  4.9 (4.3 - 5.5) |

* CGM data of three participants were not available in the control period
Day: 06:00 – 24:00
Night: 24:00 – 06:00
Data are presented as median (IQR)
Glycemic ranges and CV are presented as percentage
