# Supplementary material for: Bihormonal fully closed-loop glucose control in adolescents with Type 1 diabetes: an outpatient, randomized, crossover trial
Source: Pediatr Diabetes. 2026 Sep 2;2026:e008. doi: 10.48130/pedi-0026-0009 (PMC13599424; doi:10.48130/pedi-0026-0009)
Supplement: Supplementary file 1 [file pedi-2026-2026-1-0009-S1.zip › 10.48130_pedi-0026-0009-Suppl-FigureS1.docx]

**Bihormonal fully closed-loop glucose control in adolescents with type 1 diabetes: An outpatient, randomized, crossover trial**

**Supplementary materials**

**Supplementary Figure 1. Participant flow table.**
